# Identification of *Leishmania donovani* antigen in circulating immune complexes of visceral leishmaniasis subjects for diagnosis

**DOI:** 10.1371/journal.pone.0182474

**Published:** 2017-08-18

**Authors:** Fauzia Jamal, Pushkar Shivam, Sarita Kumari, Manish Kumar Singh, Abul Hasan Sardar, Pushpanjali, Selvasankar Murugesan, Shyam Narayan, Anil Kumar Gupta, Krishna Pandey, V. N. R. Das, Vahab Ali, Sanjiva Bimal, Pradeep Das, Shubhankar K. Singh

**Affiliations:** 1 Department of Microbiology, Rajendra Memorial Research Institute of Medical Sciences, Patna, India; 2 Department of Molecular Biology, Rajendra Memorial Research Institute of Medical Sciences, Patna, India; 3 Department of Biotechnology, National Institute of Pharmaceutical Education and Research, Hajipur, India; 4 Department of Clinical Medicine, Rajendra Memorial Research Institute of Medical Sciences, Patna, India; 5 Department of Immunology, Rajendra Memorial Research Institute of Medical Sciences, Patna, India; Meharry Medical College, UNITED STATES

## Abstract

The unreliability of most of the existing antibody-based diagnostic kits to discriminate between active and treated VL cases, relapse situation and reinfection are a major hurdle in controlling the cases of Kala-azar in an endemic area. An antigen targeted diagnostic approaches can be an attractive strategy to overcome these problems. Hence, this study was focused on identifying the *Leishmania* antigens, lies in circulating immune complex (CICs), can be used for diagnostic as well as prognostic purposes. The present study was conducted on peripheral blood samples of 115 human subjects, based on isolation of CICs. The SDS-PAGE patterns showed an up-regulated expression of 55 kDa and 23 kDa fractions in an antigens obtained from CICs of all clinical and parasitologically proven untreated visceral leishmaniasis patients before treatment (VL-BT), which ensured absolute sensitivity. However, light expressions of these bands were observed in some VL treated cases. To ascertain the prognostic value, 2D expression profiles of circulating antigens were carried out, which revealed 3 upregulated and 12 induced immunoreactive spots. Out of these, ten prominent spots were excised and subjected for enzymatic digestion to generate peptides. Mass spectrometry (MS) analysis successfully explored 20 peptides derived from kinase, kinesin, acetyl Co-A carboxylase, dynein heavy chains (cytoplasmic and axonemal/flagellar), 60S ribosomal protein, nucleoporin protein, RNA polymeraseII, protease gp63, tubulin, DNA polymerase epsilon subunit, GTP-binding protein and tyrosyl-methionyl t-RNA synthetase-like protein and 19 hypothetical protein of unknown function. Presence of *L*. *donovani* proteins in circulating antigens were further validated using anti-*Ld* actin and anti-α tubulin antibody. Besides, MS derived peptides confirmed its reactivity with patients' sera. Therefore, these shortlisted potential antigens can be explored as antigen-based diagnostic as well as prognostic kit.

## 1. Introduction

Human Visceral Leishmaniasis (VL), a lethal form of leishmaniasis caused by *Leishmania donovani* (*L*. *donovani*) contributes significantly to the annual burden of infectious diseases in India [1]. Failure in management strategies has led to the rescheduling deadline of kala-azar eradication program from 2012 to 2015 and further upto 2020.Unsatisfactory diagnosis for certain forms of VL is one of the major hurdles in a VL elimination program. The conventional diagnostic test for VL is the microscopic demonstration of *L*. *donovani* amastigotes in aspirates from visceral organ [2]. Among the parasitological diagnosis, the sensitivity of microscopic examination of lymph node, spleen and bone-marrow aspirates varies from 52–58%, 93.1–98.7% and 52–85% respectively [3]. The overall sensitivity governs by these diagnostic methods appears highly unsatisfactory, if spleenic aspiration is not considered. Additionally, aspiration of lymphoid tissue is invasive, requires expertise and crowded with complications [3]. PCR is capable of diagnosing relapse or re-infection cases of VL. But, it is complex and not suitable for mass screening in the field [3]. Serological tests like ELISA, rapid immunochromatography, direct agglutination test (DAT) etc. were although less invasive, but its reliability is not assured due to cross-reactivity in other disease conditions, co-infection cases and relapse [4]. The sensitivity and specificity of ELISA are greatly influenced by the antigen used. Whereas, in various studies, DAT has been found to be 91–100 percent sensitive and 72-100 percent specific [5]. In earlier studies, rK-39 showed 100% sensitivity and 98% specificity respectively, but it has no prognostic values as well as it is not reliable for co-infection cases and relapse cases of VL. The previous reports have also evidenced that the sensitivity of rK-39 is as low as 71% in HIV patients co-infected with *Leishmania* [4]. Further, a report from Sudan has shown the 67% sensitivity of the rK-39 test in immuno-compromised patients [6]. The Ld-rKE-16 antigen by Sa pn diagnostic ltd. Is an another successful example of commercially available rapid antibody detection antigen based on membrane filtration technology [5].

All these tests cannot discriminate between active and past infection as the IgG persists in sera even after two years of cure [3]. Since antigen levels are expected to broadly correlated with the parasite load, it is comparatively more convincing as parasite antigen is eliminated quickly from body system as soon as the disease is cured [5, 7]. Therefore, antigen detection systems may be an ideal alternative to antibody detection assay. It may also be ideal for diagnosis of immuno-compromised patients and more particularly with advanced cases of HIV co-infection, where the immune response is impaired [5, 7]. However, the efforts are far beyond satisfactory in the development of antigen targeted diagnostic tool. In 2001, a new latex agglutination test (KATEX) developed with 73.5% sensitivity and 99% specificity in detecting Leishmanial antigen in urine samples of VL patients [8]. Moreover, in our recent study B-cell epitopes RFFVQGDGIGQHSLQEALERR (P_1_) and RRVAVLVLLDRL (P_2_) from a hypothetical protein [Acc No: XP_003861458.1] of *L*. *donovani* were used as potential antigen based diagnostic as well as prognostic candidate in VL detection [9].

As it is well known that macrophages infected with *Leishmania* parasites ruptures due to hypertrophic growth and releases *L*. *donovani* in amastigotes form at the infection site. Many such amastigotes come in contact with humoral immune responses *viz*. antibody-dependent cell mediated cytotoxicity or classical pathway of complement activation. Unfortunately, a defective clearance of these immune complexes by scavenging macrophages leads their accumulation in circulation, which is known as circulating immune complexes (CICs). Previously, CICs were estimated in different diseases using the platelet aggregation test [10], complement deviation test [11–14] and polyethylene glycol precipitation test [15, 16]. Further, Evans and Pearson [17] suggested that the identification of circulating parasite antigen could have potential diagnostic and prognostic value. The CICs are detectable in a variety of systemic disorders such as rheumatologic and autoimmune disease [18–22], allergic disease [23–25], infectious diseases [26–34], hormonal disease [35], during pregnancy [36–38] and various other cases, which may induce glomerulonephritis [39–41]. Therefore, *Leishmania* antigens present in CICs may also be explored for diagnosis.

This study was designed to identify *Leishmania* antigen in CICs for the diagnosis of different clinical stages of *L*. *donovani* infection like fresh VL cases (VL before anti-Leishmanial therapy), treated cases (patient received a full course of treatment and declared clinically cure), relapse (re-emergence of *L*. *donovani* in VL treated cases within six months after anti-leishmanial therapy) and re-infection (Re-infection of *L*. *donovani* in cured VL cases after six months from the end of anti-leishmanial therapy). The present data reveals that CICs in VL patients carries some *Leishmania*-specific antigen fragments, which may have good diagnostic as well as prognostic value.

## 2. Material and method

### 2.1. Sample selection

Approval and recommendations were obtained from the Institutional Ethics Committee (IEC) of the Rajendra Memorial Research Institute of Medical Sciences, Indian Council of Medical Research, India, for the written informed consent form (printed in the language of donor), sample size and procedures as per Helsinki declaration. Left over serum samples of patients (of both sex and age groups, 5 to 45 years) were collected during the diagnosis process at an institute OPD after getting written informed consent from the patient or from next to kin or guardians in case of minors in the presence of a witness signatory. Blood sample of healthy subjects was pooled directly for this purpose after getting the written informed consent. Total sample (n = 115) includes 50 active VL cases (VL-BT that represents fresh VL plus reinfection/ relapse cases), of which, patients were followed up to the end of amphotericin B treatment (15 injections of 1 mg/kg body weight applied with very slow infusion of 5% dextrose on alternate days) (VL-AT). Besides, blood from 25 other diseased cases (comprising 10 tuberculosis cases with positive sputum culture, 5 microfilariae positive cases of lymphatic filariasis, 5 clinically confirmed cases of asthma having chronic airway hyper responsiveness, 2 cases of dengue positive to ELISA, 2 cases of Japanese Encephalitis positive to ELISA and one case of influenza A positive to ELISA) and 40 healthy subjects (25 endemic and 15 non-endemic) were also collected. Samples were collected from sera bank and OPD of RMRIMS after getting informed consent. Each healthy subject had no apparent history of VL or other disease in recent past. These sera samples were used for evaluation of concentration of CICs. VL subjects were diagnosed parasitologically [42] at Rajendra Memorial Research Institute of Medical Sciences, Patna. 2 ml peripheral blood samples were collected from above 40 VL-BT, 10 VL-AT, 10 other diseases and 10 healthy subjects for isolation of antigen from CICs and study through sodium dodecyl sulphate polyacrylamide gel electrophoresis (SDS-PAGE). Six samples of VL-BT and VL-AT were studied through two-dimensional electrophoresis.

### 2.2. *L*. *donovani* culture and isolation of SLA

Soluble *Leishmania* antigen (SLA) was included in this study as a control. For this, a reference strain of *L*. *donovani* (MHOM/IN/83/AG83; repository number: RMRI/PB-0078) was used for preparation of SLA. The Promastigote culture was maintained as described elsewhere. Further, late log phage parasites were incubated at 30°C and 37° C for two hours in CO_2_ incubator [43, 44]. The cultures were centrifuged in 50 ml centrifuge tubes (Tarson, India) at 840 × g for 20 minutes at 4°C in a cooling centrifuge (Hermle, Germany). The pellet was washed twice with PBS (Phosphate buffer saline) by centrifuging at 840 × g for 20 minutes at 4°C followed by six freezing and thawing cycle. The lysate was centrifuged at 30,000 × g for 30 minutes and the supernatant was collected in aliquots and stored at —80°C for further use.

### 2.3. Isolation of CICs

The Peripheral blood was collected in serum collection tube and allowed to coagulate in slanting position for 2 hours at room temperature (RT). After coagulation, the blood samples were centrifuged at 500 × g for 15 minutes. Sera supernatant (free from RBCs) was collected in vials. Serum was precipitated in buffer containing 5% Polyethylene glycol (PEG, Merck, India) and 0.1M Sodium borate (Sigma), pH 8.5 for overnight at 4°C [34]. The precipitated CICs was isolated by centrifugation at 6000 × g for 45 minutes. The pellet was further washed for three times at 6000 × g in 2.5% PEG containing borate buffer (0.1 M) at 4°C for three times, dissolved in Dulbecco’s Phosphate Buffered Saline (Sigma), pH 7.2 and stored at -80°C in aliquots for further use. The protein content of isolated CICs was estimated with protein estimation kit (Merck Biosciences) based on Lowry’s method [45].

### 2.4. Acid dissociation of CICs and removal of antibody using staphylococcal protein A agarose

Acid dissociation of antigen and antibody present in CICs was performed using the glycin-HCl buffer at pH 2.5–2.8 (to obtain final pH 3) as described by Gupta and Tan [46]. Further, it was incubated with equilibrated protein A agarose (Sigma), at RT on shaker incubator for 45 minutes. The mixture was centrifuged at 500 × g for 10 minutes. The antigen free from antibody was obtained from the supernatant due to adsorption of antibody fraction on swollen protein A agarose. Supernatants were dialyzed using Micro Dispo Dialyzer^TM^ apparatus, (Pall Life Sciences, India) against three changes of 300 ml PBS for 24 hours at 4°C. Immunoglobulin bounded protein A agarose (present in pellet) was washed twice with PBS at 840 × g for 15 minutes. Washed pellet was incubated in 2 ml (or twice with the volume of pellet) of 3.5 M MgCl_2_ for 15 minutes, at RT followed by centrifugation at 500 × g for 10 minutes. Supernatant containing antibody was dialyzed and washed as described above. Protein concentration was evaluated by Lowry method [45].

### 2.5. Immunoprecipitation, antibody isolation and HRP conjugation

**Fig 1:**
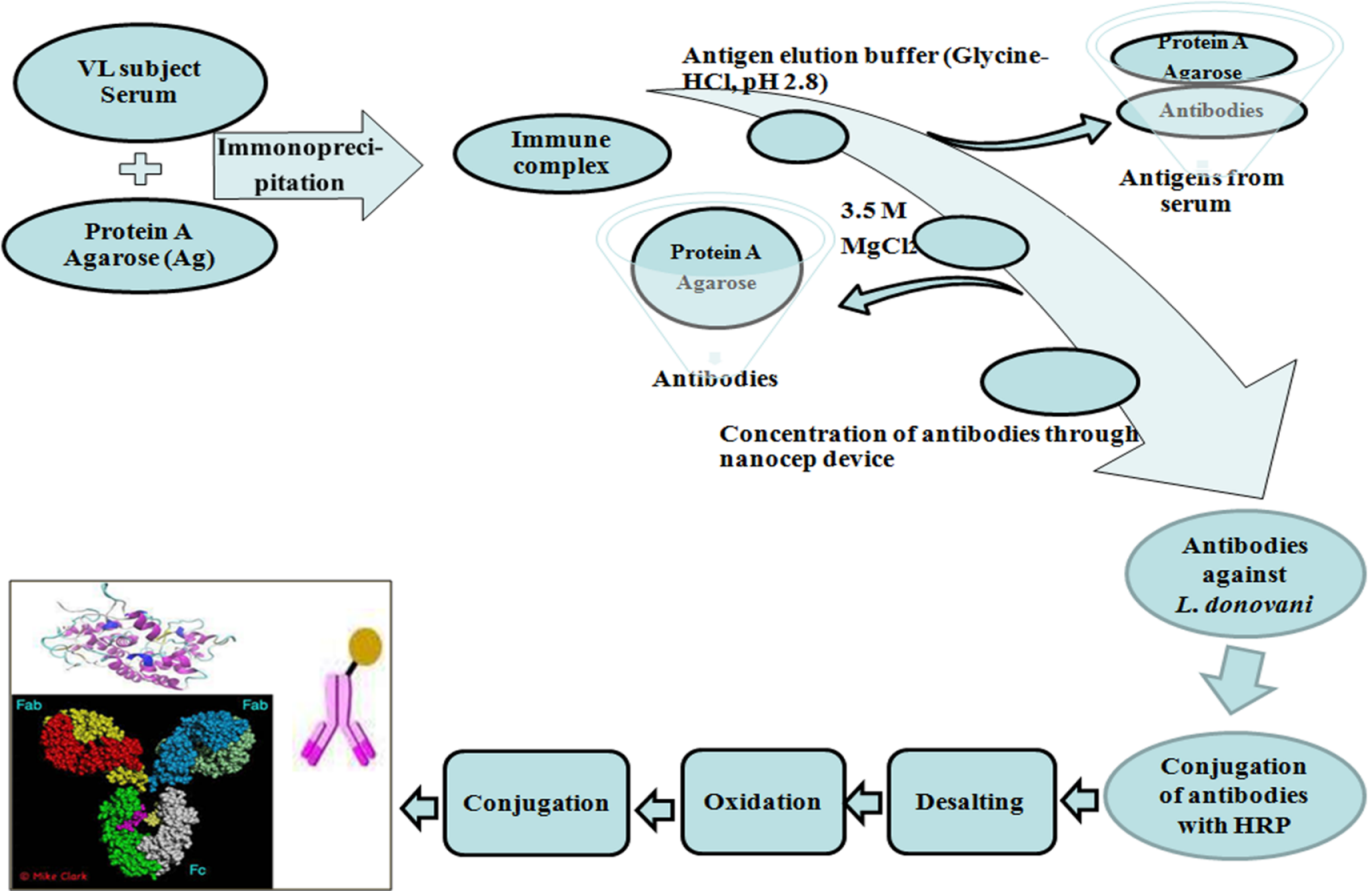
Flow diagram showing procurement and HRP labeling of anti-leishmanial primary antibody. Blood serum was isolated from parasitologically confirmed VL patient sera. Anti-leishmanial antibodies present in VL-BT was immunoprecipitated with protein A agarose. MgCl_2_ was used to dissociate VL specific antibody. Eluted antibody was tagged with and HRP using HRP labeling kit to prepare Kala-azar antigen detection antibody.

The antibody of confirmed Kala-azar (VL-BT) subject was immunoprecipitated with Protein A agarose (Sigma) (Fig 1). Antigen was eluted from antigen-antibody-protein A agarose tri-complex in a column with the help of glycin-HCl buffer followed by washing with PBS. Antibody-protein A agarose complex was dissociated using 3.5M MgCl_2_ solution and eluted from the column. Further, the antibody was passed through Nanosep^®^ centrifugal device (Pall Corporation) for concentrating and desalting the sample. Finally, the concentrated and purified antibody was dissolved in PBS. This antibody was labelled with (horse reddish peroxidase) HRP by using HRP labeling Kit (Bangalore Genei, India).

### 2.6. SDS-PAGE and immunoblotting of CICs antigen resolved by SDS-PAGE

50μg CICs antigen of different individuals of different study groups was electrophoresed in discontinuous SDS-PAGE. For this, 10% resolving gels were prepared with 30% acrylamide and bis-acrylamide solution, 1.5 M Tris-HCl (pH-8.8), 10% SDS, 10% APS, and 5 μl TEMED. The electrophoresis were done in mini Protein III cell (BioRad, USA) using electrophoresis buffer (tris 0.025 M, glycin 0.19 M and SDS 0.1%) by following the protocol of Laemmli [47]. Further, the gels were stained with 0.25% Coomassie brilliant blue R250 and analyzed in comparison to standard Marker [Fermentas (broad range), USA]. The Image of the gel and nitrocellulose paper was captured by Gel Dock (Bio-Rad, USA). The molecular weight of polypeptides was calculated by using standard protein molecular weight marker on Quantity One software (Bio-Rad, USA). Western blotting was performed using HRP conjugated anti-leishmanial antibody (antibodies isolated from VL-BT patients, conjugated with HRP using HRP labeling kit, Bangalore Genei, India) following the protocol of Singh and his colleagues [43].

### 2.7. 2D gel electrophoresis and mass spectrometry compatible silver staining

The two-dimensional gel electrophoresis was performed with Ready prep^TM^2D starter kit (Bio-Rad). Antigen samples were purified by micro-bio-spin chromatography column (Bio-Rad) to remove salts. 40 μg of protein were run on 7 cm IPG strip (pH 3–10, 7 cm, Bio-rad) according to protocol mentioned in ready prep ^TM^2D starter kit in PROTEIN IEF cell in step wise fashion viz. step1: 250V, linear for 20 minutes; step 2: 4,000V, linear for 150 minutes; step 3: VH-10000, rapid; step 4: Hold Step-500V for 4 hrs. The IPG strip was subjected to SDS-PAGE (2^nd^ Dimension). 12% resolving gel was prepared and poured in the Bio-Rad gel cast. IPG strip was laid onto the polyacrylamide gel bed. It was electrophoresed in Tris-glycine SDS-PAGE running buffer (1X) at constant 200V for 40 minutes. Silver staining of the electrophoresed gel was performed using Plus One Silver Staining Kit (GE healthcare) following manufacturer’s manual. MS compatible staining procedure for gel was adapted from Plus One Staining Kit manual (Amersham). By omitting the glutaraldehyde from the sensitizer and formaldehyde from the silver solution, the method becomes compatible for mass spectrometry analysis, however, at the expense of sensitivity (10 ng). Therefore, only the prominent spots were carefully excised from the gel.

### 2.8. De-staining and tryptic digestion of silver stained spots

Silver stained spots were excised from 2D gel and dried. To enrich protein samples, spots from three gels in triplicate were pooled. Excised spots were destained in solution containing 30 mM potassium ferricyanide and 100 mM sodium thiosulfate solution (1:1), twice by incubating for 10 minutes each. Further, destained gel spots were washed twice for 1 hour each with water, followed by 10 minutes washing with 25 mM ammonium bicarbonate in acetonitrile (ACN) and water in the ratio of 1:1. The excised gel was vacuum dried [48] and subjected for digestion in 10 μl trypsin containing solution (12.5 mg/ml trypsin, 25 mM ammonium bicarbonate) and incubated overnight at 37°C [47]. The digested polypeptide was extracted using 20 μL ACN containing 1% Trifluoroacetic acid (TFA). The mixture was incubated twice for 20 minutes and pooled extract was dried for further use.

### 2.9. HPLC-MS and identification of peptides

Prior to MS, different peptides present in polypeptide solution were fractionated with HPLC. Peptides were analyzed by electrospray ionization mass spectrometry using the Ultimate 3000 nano-HPLC system [Dionex] coupled to a 4000 QTRAP mass spectrometer [Applied Biosystems]. Tryptic peptides were loaded onto a C18 PepMap100, 3 μm [LC Packings] and separated with a linear gradient of water/acetonitrile/0.1% formic acid (v/v). Further, spectra were analyzed to identify proteins of interest using the following parameters: database; Ludwig NR database; Mascot sequence matching software [Matrix Science] with Uniprot Leish PR database, Type of search; MS/MS Ion Search, Enzyme; Trypsin, Variable modifications; Oxidation (M), Mass values; Monoisotopic, Protein Mass; Unrestricted, Peptide Mass Tolerance; ± 1.2 Da, Fragment Mass Tolerance; ± 0.6 Da, Max Missed Cleavages; 1 and Instrument type; ESI-TRAP.

### 2.10. Validatory evaluation of the presence of *L*. *donovani* antigen in CICs

To validate the presence of *L*. *donovani* antigen in circulation 50μg CICs, antigen of different individuals of different study groups was electrophoresed in discontinuous SDS-PAGE. Western blotting was performed with anti-*Ld* actin antibody (kind gift from Dr. Amogh A. Sahasrabuddhe, CDRI, Lucknow, India), which were used at 1:5000 dilution, whereas alkaline phosphatase-conjugated goat anti-rabbit IgG antibody (Santa Cruz) was used at 1:2000 dilution. The blots were visualized using nitro blue tetrazolium/5-bromo-4-chloro-3-indolyl phosphate solution (Santa Cruz) according to the manufacturer’s recommendations. Presence of tubulin was displayed with the help of anti-tubulin antibody following protocol of Sardar *et al*. 2013 [49].

To validate the MS derived peptide sequence, representing B cell epitopes of *L*. *donovani*proteins XP_003861271.1 (RFFVQGDGIGQRSLQEALERRVAVLVLLDR) and XP_003861300.1 ARNELYDMLEIDPPAARAANAGESANE were synthesized commercially [9]. Spot ELISA were performed to validate the presence of antibody against these peptides in VL patients. 0.5 μg of SLA and synthetic peptide in PBS were loaded as a dot on ELISA plate coated with Nitro cellulose paper (NCP) for overnight, following protocol of Jamal *et al*. (2016) [9]. As positive and negative control, SLA and PBS was used. Blocking was done with 5% BSA in PBS, for 2 hours followed by three washing with TBS-T. TBS-T with 0.2% BSA and 0.1% tween-20 was used as wash buffer. Experiment was performed in two different sets. In first set PBS, SLA and peptides were incubated with VL sera, in another set with healthy sera, for two hours followed by three washing with TBS-T. HRP tagged anti-human IgG was used as secondary antibody (Merck biosciences), incubated for 1 hour followed by three washing with TBS-T. DAB was used as substrate for detection.

### 2.11. Statistical analysis

Statistical analysis was performed using Graph Pad Prism 6. One way ANOVA was performed to evaluate the statistical significance of the obtained data on CICs concentration and densitometry of bands in SDS-PAGE and NCP.

## 3. Result

### 3.1. Higher accumulation of CICs in human VL subjects during *L*. *donovani* infection

**Fig 2:**
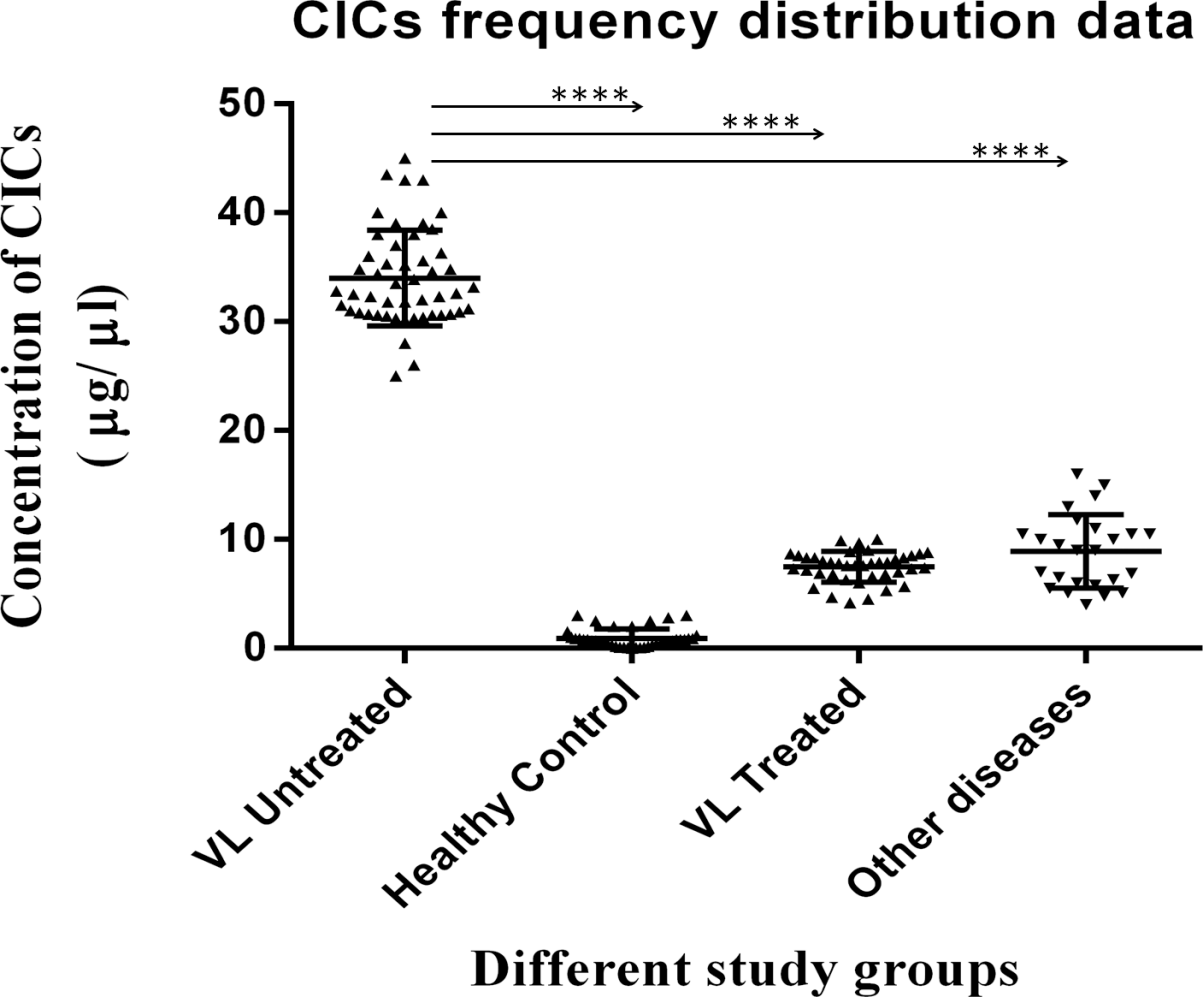
Figure showing accumulation of CICs in human VL subjects during *L*. *donovani* infection in comparison to others. For this, one ml of peripheral blood serum was taken from the different study groups. It was incubated with precipitation buffer (5% polyethylene glycol and 0.1 M sodium borate) for overnight. The precipitated CICs was washed with wash buffer containing 2.5% PEG 6000 and re-dissolved in 100 μl PBS. Protein content (in μg/μl) was evaluated using Lowry method. 16 μg/μl was evaluated as cut-off concentration analyzed using graph pad prism 6.0.

The concentrations of CICs in samples from VL subjects are depicted in Fig 2. The graph Pad Prism 6.0 data revealed 16 μg/μl as a cut-off concentration of CICs. Overall, a 35 fold higher concentration of CICs was observed in VL-BT subjects, which reduced significantly in samples from VL-AT (P = 0.0001). The reduced CICs concentration in VL-AT subjects was even eight folds higher than healthy subjects. Besides, we also identified an elevated CICs in samples from other disease in comparison to control but the overall increase was towards a much higher side in VL-BT subjects (P = 0.0001).

### 3.2. Up-regulated expression of CICs antigen in samples from VL subjects

**Fig 3:**
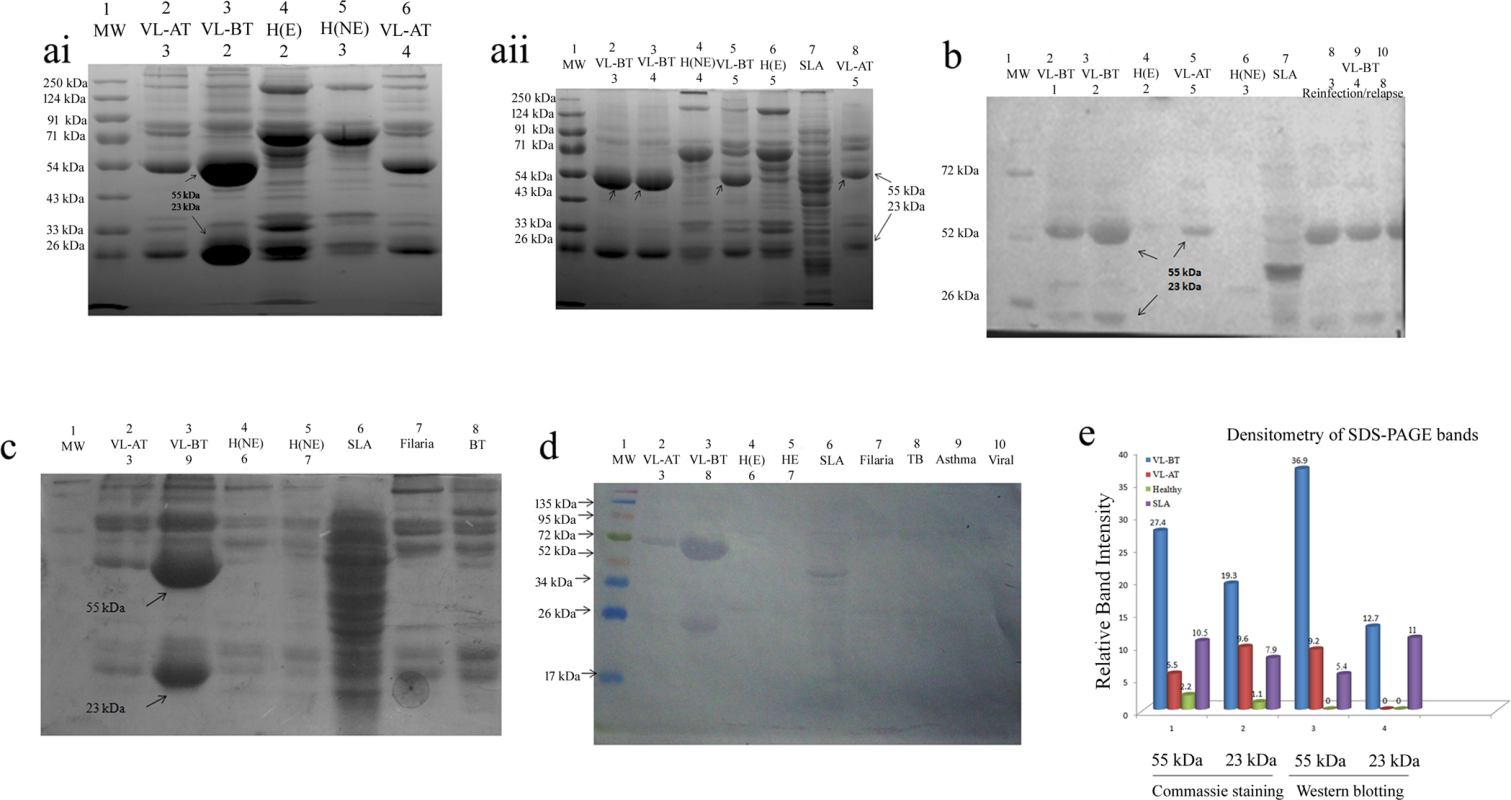
SDS-PAGE showing the banding pattern of electrophoresed CICs antigens in VL-BT subjects compared to healthy, treated VL, relapse, reinfection and other diseases subjects. Briefly, 50μg of proteins was electrophoresed (10% SDS-PAGE) and stained with Coomassie Brilliant Blue R-250 or immunoblotted. **Fig** 3.a.i. SDS-PAGE comparing VL-BT subjects to healthy and treated VL cases. Molecular weight marker in lane 1; CICs antigen of VL-AT samples in lane-2 & 6,CICs antigen of VL-BT samples in lane-3 and healthy endemic and non-endemic in lane 4 and 5. VL-BT samples are expressing up-regulated 55 and 23 kDa antigen (indicated by ↑). VL-AT (lane 2 & 6) is also showing comparatively down-regulated expression of 55kDa and 23kDaband. Fig 3.a.ii. SDS-PAGE showing the banding pattern of electrophoresed CICs antigens in VL-BT (relapse/reinfection) subjects compared to healthy and treated VL cases and SLA. Molecular weight marker in lane 1; CICs antigen of VL-BT samples in lane-2,3 (Reinfection/relapse) & 5; healthy endemic and non-endemic in lane 4 and 6; SLA in lane 7 and VL-AT in lane 8. VL-BT samples are expressing up-regulated 55 and 23 kDa antigen (indicated by ↑). VL-AT (lane 8) and healthy endemic (lane 4) is also showing comparatively downregulated expression of 55 and 23kDaband.Fig 3.b. Immunoblotting data of SDS-PAGE (the gel of Fig 3.a.) showing immunoreactive bands in different study groups. Electrophoresed gel was transferred to NCP membrane and exposed to anti-leishmanial antibody HRP conjugated followed by substrate (DAB) exposure. Molecular weight marker in lane 1;CICs antigen of VL-BT samples in lane-2,3 and 8–10;VL-AT in lane 5; SLA in lane 7 and healthy in lane 4 and 6. 55 and 23 kDa can be recognized in all VL-BT samples (indicated by ↑). VL-AT (lane 5) is also showing comparatively downregulated expression of 55 kDa band. 23 kDa band is not recognizable in this sample. Fig 3.c. SDS-PAGE showing the banding pattern of electrophoresed CICs antigens of VL-BT subjects in comparison to healthy and treated VL cases and other diseases. Molecular weight marker in lane 1;CICs antigen of VL-AT samples in lane-2;VL-BT in lane 3; healthy endemic and non-endemic in lane 4 and 5; SLA in lane 6; Filaria in lane 7 and TB in lane 8. VL-BT samples are expressing up-regulated 55 and 23 kDa antigen (indicated by ↑) in comparison to others. Fig 3.d. Immunoblotting data of SDS-PAGE (the gel of Fig 3.c.) showing immunoreactive bands in different study groups. Molecular weight marker in lane 1;CICs antigen of VL-AT samples in lane-2;VL-BT in lane 3; healthy in lane 4 and 5; SLA in lane 6; Filaria in lane 7, TB in lane 8, Asthma in lane 9 and viral in lane 10. VL-BT samples are expressing up-regulated 55 and 23 kDa antigen (indicated by ↑) in comparison to others except low intensity band at 55kDa band appeared in VL-AT. Fig 3.e. Histogram showing comparative band intensity of 55 and 23 kDa SDS-PAGE gel and after immunoblotting. Relative band intensity was evaluated using Quantity one software. Band intensity of SLA in stained gel and after blotting was revealed as 10.5, 7.9 and 5.4, 11 respectively, confirmed the presence of the protein of similar molecular weight in *L*. *donovani*.

The antigens isolated from CICs of different study groups were electrophoresed in 10% SDS-PAGE to evaluate the molecular weight of polypeptide fractions (Fig 3A.i, Table 1). Multiple bands of molecular weight 100kDa, 90kDa, 70 kDa, 65kDa, 63kDa, 62kDa, 55 kDa, 45 kDa, 42 kDa, 41kDa, 37 kDa, 31.6 kDa, 30kDa, 28 kDa, 23 kDa, 21kDa and 19 kDa were observed in SLA (Lane 7, Fig 3A.ii & 3C). The fourteen peptide fractions of CICs antigen was common between VL-BT and VL-AT subjects (182 kDa, 100kDA, 90kDa, 82kDa, 65 kDa, 62 kDa, 55 kDa, 42 kDa, 37 kDa, 31.6kDa, 30 kDa, 28kDa, 23kDa, 21 kDa). Except 182 kDa and 82 kDa bands, all bands identified in CICs were also present in SLA. One additional fragment of 36 kDa was observed in sample of tuberculosis patient (Fig 3C). Similarly, there was an additional band of 92 kDa in CICs antigen of Asthma. In addition to the above, there were some sporadic bands in some of the samples; all bands explained above were reproducible within the study group. Notably, a dominant expression of 55 kDa and 23 kDa in CICs antigen of VL-BT was not observed in VL-AT (Fig 3A and 3C). Densitometry analysis (Quantity One software, Bio-Rad) revealed differences in relative quantity of bands (Fig 3E). Relative quantities of 55 kDa band were 27.4, 5.5 and 10.5 in VL-BT, VL-AT and SLA respectively. Likewise, relative quantities of 23 kDa band were 19.3, 9.6 and 7.9 in VL-BT, VL-AT and SLA respectively. However, the cutoff in term of the relative intensity of bands for these two antigens 55 kDa and 23 kDa were 2.2 and 1.1 respectively.

**Table 1:** SDS-PAGE fractions of CICs antigen isolated from different study groups. The molecular weight of bands in Kilodalton calculated from SDS-PAGE of different study groups. The bands were measured using Quantity One software (BioRad). ‘+’ indicate down regulation of bands.

SDS-PAGE band in kDa	*L*. *donovani* antigen	Antigen from CIC of Healthy Subject			Antigen from CIC of Other Diseased Subject					
	SLA	VL-BT	VL-AT	Healthy (VL endemic)		Healthy (VL Non-endemic)	TB	Asthma	Viral infection	Filaria
182		+	+	+		+	+	+	+	+
100	**+**	**+**	+			+				
92								+		
90	**+**	**+**	+	+		+	+	+	+	
82		+	+	+		+	+	+	+	+
70	+									
65	**+**	+	+	+		+	+	+	+	+
63	+									
62	+	+	+	+		+	+	+	+	+
55	**+**	**+**	+	+		+	+	+	+	+
45	+									
42	+	+	+	+		+	+	+	+	+
41	**+**									
37	+	+	+				+	+	+	+
36							+			
31.6	+	+	+	+		+	+	+	+	+
30	+	+	+	+		+	+	+	+	+
28	+	+	+	+		+	+	+	+	+
23	**+**	**+**	+	+		+	+	+	+	+
21	+	+	+	+		+	+	+		+
19	+									

### 3.3 Many of the antigenic fractions isolated from CICs were immunoreactive in Western immunoblotting

Out of the total antigenic fragments, obtained of SLA in SDS-PAGE (90 kDa, 70 kDa, 63 kDa, 62 kDa, 55 kDa, 45 kDa, 41 kDa, 31.6 kDa, 30kDa, and 23 kDa) Eight polypeptides showed immunoreactivity to anti-*L*. *donovani* primary antibody (Fig 3B). Amongst them, three polypeptides (55 kDa, 37kDa and 23 kDa) were immunodominant. Especially, the 55 kDa and 23 kDa polypeptide displayed reactivity in all VL-BT samples (Fig 3B). The specificity in them was confirmed through a negative reaction observed in the sample of asthma, tuberculosis, filarial, viral flu etc. (Fig 3D, lane 6–9). Comparatively, a less immunoreactive band of 62 kDa was also observed, which showed its immunoreactivity to anti-*L*. *donovani* primary antibody in samples of another disease. A sporadic band of 37 kDa was also observed which was marked as reactive in few samples of VL-BT and healthy subjects, but its sensitivity was less. Except in two samples, 23 kDa band was not recognizable but 55 kDa band was observed with 4.9 fold less band intensity in VL-AT samples.

The Relative quantity of 55 kDa and 23 kDa bands in Western blotting in the given figure were 36.9 and 12.7 in VL-BT subjects in comparison to SLA (Fig 3E), which was 5.4 and 11 respectively. Thus, the immunoreactivity established *L*. *donovani* as a source of these proteins. The Relative density of 55 kDa band in VL-AT sample was 9.2 (down-regulated). Therefore, we identified a strong discriminative ability of both the polypeptide between active and treated cases of VL.

### 3.4. Many specific and immunoreactive spots of VL-BT samples were observed in 2D resolution and immunoblotting

**Fig 4:**
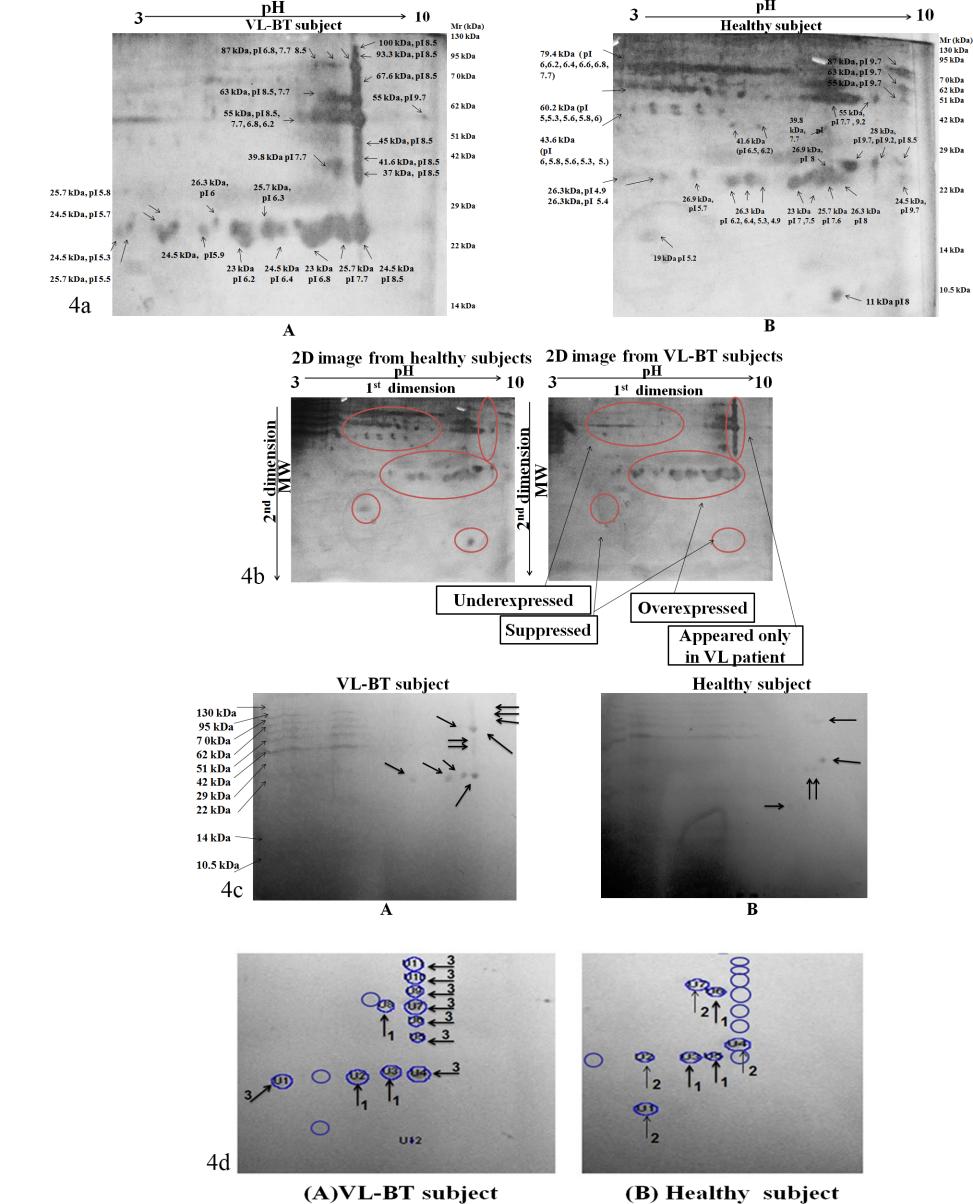
Figure showing 2D gel electrophoresis data. 40μg of proteins was subjected to IPG strips (3–10 pH, 4–7 cm, non-linear) in the first dimension followed by SDS-PAGE (12%) in the second dimension. (a). Silver stained 2D spots are visualized in the sample from VL-BT subject (A) and healthy subject (B). The number of 2D spots was more in healthy subjects whereas in VL-BT intense and specific 2D spots were observed. (b). Figure showing differences in different regions of the gel and exclusive expression of spots. (c). Figure showing immunoblot of the gel of Fig a.(A) and (B). The sample of VL-BT subject showed 100 kDa, 93.3 kDa, 87 kDa, 67.6 kDa, 63 kDa, 55 kDa, 45.7 kDa, 41.6 kDa, 37 kDa all having pI 8.5 as well as 24.5 kDa (pI 8.5), 23 kDa (pI 6.8, 6.2) immunoreactive spots. Besides, 55 kDa (pI7.7) 26.3 kDa (pI 8) and 25.7 kDa (pI 7.7) dots were upregulated in the immunoblot of the sample from VL-BT subjects in comparison to the sample from healthy subjects. (d). Figure showing comparative densitometry analysis of immunoreactive spots. Densitometry data of immunoreactive 2D spots of VL-BT and healthy subject after western immune-blotting with anti-*Leishmania* antibody isolated from patients are given in term of volume (INT*mm2), and area (mm^2^). Immuno-reactive 2D spots present in both VL-BT and healthy are indicated in **↑**_**1,**_ ↑_**2**_ indicates 2D spots only present in the healthy subject and 2D spots only present in VL-BT subject indicated by **↑**_**3.**_

Distinct silver stained spots were identified in whole antigen content isolated from CICs of VL-BT subject and healthy subject. Consecutive runs produced identical 2D pattern. Thus, the reproducibility was confirmed and considered final. The 2D pattern of excised and isolated 55 kDa and 23 kDa bands were similar to a segment of whole antigen 2D (S1 and S2 Fig). Since 2D spot pattern in VL-AT gel was almost alike to that of healthy (S3 Fig), the result was compared with the 2D gel of only healthy subject. The whole antigen was segregated into 46 protein spots. The range of predicted molecular mass of identified proteins was from 20 to 125 kDa. A majority of proteins exhibited a molecular mass between 37 to 87 kDa. Likewise, well resolve protein spots were detected within pI range of 3–10. A majority of these spots were detected around pI 6 to 8.5. The molecular weight of antigenic2D spots in VL-BT and healthy subjects has specified in Fig 4a.A & 4a.B (S1 Table).

Differences were identified on the basis of up-regulation, down-regulation, appearance only in patient and repression of spots (Fig 4B). To achieve this, the densitometry of 2D spots were performed (Panels A and B in S4 Fig; S2 Table). Twenty 2D spots were found in VL-BT subjects that were absent from healthy subject sample, *viz*. 100 kDa, pI 8.5, 93.3 kDa, pI 8.5, 87 kDa, pI 8.5, 67.5 kDa, pI 8.5, 63 kDa, pI 8.5, 45 kDa. pI 8.5, 41.6 kDa, pI 8.5, 37 kDa, pI 8.5, 26.3 kDa, pI 6, 25.7 kDa, pI 7.7, 6.3, 5.8, 5.3, 24.5 kDa, pI 8.5,6.4, 5.9, 5.7, 5.3 and 23 kDa, pI 6.8, 6.2. Eighteen 2D spots were found suppressed in the case of VL-BT subject in comparison to the healthy subject, *viz*. 87kDa, pI 9.7, 63 kDa, pI 9.7, 55 kDa, pI 9.2, 41.6 kDa, pI 6.5, 6.2, 28 kDa, pI 9.7, 9.2, 8.5, 26.9 kDa, pI 8, 5.7, 26.3 kDa, pI 7.3, 6.4, 6.3, 25.7 kDa, pI 7.6, 24.5 kDa, pI 9.7, 19 kDa, pI 5.2, 15 kDa, pI 7.7 and 11 kDa, pI 8. Five 2D spots, which were found up-regulated in VL-BT subject in comparison to healthy subjects, were 55 kDa, pI 8.5, 7.7, 39.8 kDa, pI 7.7, 26.9 kDa, pI 8.3 and 25.7 kDa, pI 7.6. Fourteen 2D spots *viz*. 79.4 kDa, pI 7.7, 6.8, 6.6, 6.4, 6.2, 60.2 kDa, pI 5.8, 5.6 5.3,5 and 43.6 kDa, pI 6, 5.8, 5.6, 5.3, 5 were less prominent in VL-BT in comparison to healthy subjects. Four 2D spots (55 kDa, pI 9.7, 26.9 kDa, pI 8.3 and 26.3 kDa, pI 5.4, 4.9) were consistently present in all samples of VL-BT and healthy subjects.

Western blotting of 2^nd^ dimension gel of VL antigenic fraction and healthy antigenic fraction showed several differences in spots after immunoblotting with the anti-leishmanial antibody (Fig 4C). The immunoreactive antigenic spots of molecular weight were 100 kDa, 93.3 kDa, 87 kDa, 67.6 kDa, 63 kDa, 55 kDa, 45.7 kDa, 41.6 kDa, 37 kDa all having pI 8.5 as well as 55 kDa (pI 7.7), 26.3 kDa (pI 8), 25.7 kDa (pI 7.7), 24.5 kDa (pI 8.5), 23 kDa (pI 6.8, 6.2) in VL-BT subjects (Fig 4C. A). In contrast, cross-reactive antigenic 2D spots in healthy subjects were 63 kDa (pI 7.6), 55 kDa, (pI 7.7), 28 kDa (pI 8.5) and 26.3 kDa (pI 8), 25.7 (pI 8.5, 7.7) and 15 kDa pI 7.7 (Fig 4C and 4B). The relative density of immunoreactive 2D protein spots (U1 to U12) in VL-BT and (U1 to U7) in healthy subjects are depicted in Fig 4D.A & 4D.B (S3 Table).

### 3.5. Antigenic peptides of Leishmanial proteins were identified in 2D spots by LC-ESI-MS/MS

**Fig 5:**
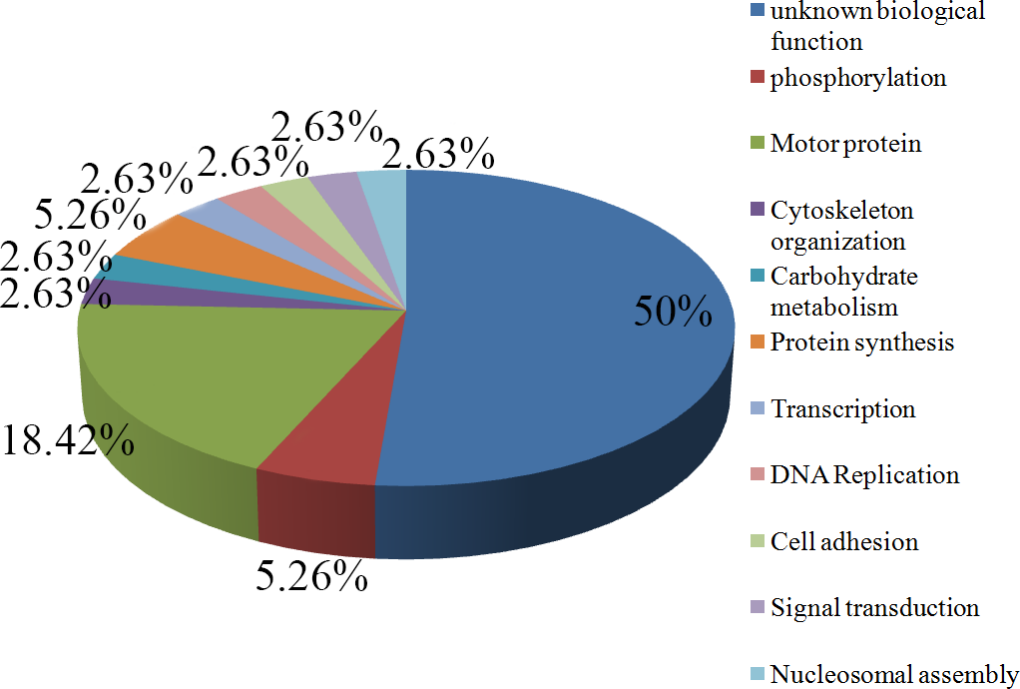
Pie chart representing the percentage and biological functional categories of ESI-LC-MS/MS identified antigen isolated from CICs (based upon their putative functions assigned using protein function database). Hypothetical protein was abundant with maximum coverage (50%). Motor proteins are second abundant protein. Protein of phosphorylation ant protein synthesis was third most abundant protein. Proteins of cytoskeleton organization, carbohydrate metabolism, transcription, DNA replication, Cell adhesion, signal transduction and nucleosome assembly are present as minor proteins.

2DE spots of 93.3 kDa, 87 kDa, 63 kDa, 55 kDa, 45 kDa, 37 kDa, 24.5 kDa all at pI 8.5, 25.7 kDa (pI 7.7) kDa and 23 kDa (pI 6.2 and 6.8) (spot A-J), which were prominent immune-reactive, were further selected for LC-MS for in-depth characterization. In total, 46 well-resolved spots of varying intensity were detected in the silver-stained gel (Fig 4A). Among them 13 spots were immunoreactive. Ten spots were exclusively immunoreactive in VL-BT. 39 proteins have been identified from VL-BT antigens, which are very noteworthy proteins. The mass spectra of peptides obtained from each spot provided sufficient signals to search Databases Matrix Science. The Proteome specific information of identified proteins, such as accession number, molecular mass, pI, and the number of peptides matched is mentioned in Table 2. Significant protein hits were obtained in each spot. Proteins identified during LC-ESI-MS/MS were associated either with characterized proteins or hypothetical conserve protein or hypothetical proteins of unknown function (S1 Dataset). The Peptides of cytosolic dynein heavy chain were predominant in the spot I and spot J. On the basis of their biological function, identified proteins were classified into 11 groups (Fig 5). Fifty percent of identified protein was the hypothetical protein of unknown function. Most of the known protein was concerned with the physio-pathological function of *L*. *donovani*. Motor protein was the second largest group (18.42%) of the identified protein, whereas protein involved in phosphorylation and synthesis (5.26%) was the third largest group. Protein involved in cytoskeleton organization, carbohydrate metabolism, transcription, DNA replication, cell adhesion, signal transduction and nuclear assembly were remaining functional category of identified protein reported in this work.

**Table 2:** Table showing identified proteins of *L*. *donovani* by ESI-LC-MS/MS analysis. List of identified proteins of *L*. *donovani* showing apparent molecular mass of peptide in kDa, pH at end of migration, identified protein, spot number, accession number (NCBI), protein hits molecular mass, number of antigenic peptides of protein present in spot.

Serial No.	Apparent molecular mass in kDa	pH at end of migration	Identified Protein	Spot	Accession No. (NCBI)	Hits Molecular mass (kDa)	No. of antigenic peptides of protein present in spot
1	93.9	8.5	Protein Kinase, putative	A	XP_003862776.1	98	3
2	93.3	8.5	Acetyl Co-A Carboxylase, putative	A	XP_003863393.1	241	5
3	87	8.5	Uncharacterized protein, Conserved	B	XP_003864769.1	78	6
4	87	8.5	Dynein heavy chain, cytosolic, putative	B	XP_003860841.1		3
5	87	8.5	Kinesin, putative	B	XP_003861238.1	343	3
6	87	8.5	Uncharacterized hypothetical protein, Conserved	B	XP_003857950.1	399	4
7	87	8.5	Hypothetical protein, unknown protein function	B	XP_003860226.1	29	3
8	87	8.5	Putative 60 S ribosomal protein	B	XP_003864802.1	17.5	2
9	63	8.5	Hypothetical protein, Conserved	C	XP_003861271.1	42	2
10	63	8.5	Hypothetical protein, Conserved	C	XP_003864131.1		4
11	63	85.	Hypothetical protein, Conserved	C	XP_003861458.1	232	4
12	63	8.5	Hypothetical protein, Conserved	C	XP_003859765.1	603	4
13	63	8.5	Hypothetical protein, Conserved	C	XP_003859244.1	218	3
14	63	8.5	Dynein heavy chain, axonemal, putative	C	XP_003864528.1		6
15	63	8.5	DNA directed RNA polymerase II subunit 2	C	XP_003863107.1	133	2
16	55	8.5	Hypothetical protein, Conserved	D	XP_003860430.1	29	2
17	55	8.5	Hypothetical protein, Conserved	D	XP_003860609.1	347	2
18	55	8.5	Kinesin, putative	D	XP_003864326.1	117	1
19	45	8.5	Hypothetical protein, Conserved	E	XP_003858545.1	261	2
20	45	8.5	Hypothetical protein, Unknown function	E	XP_003865007.1	620	3
21	45	8.5	Tubulin folding cofactor D, putative	E	XP_003861300.1		4
22	45	8.5	Major surface protease gp63, putative	E	XP_003862159.1	60.5	3
23	45	8.5	Hypothetical protein, Conserved	E	XP_003861470.1	105	3
24	45	8.5	Hypothetical protein, Conserved	E	XP_003858891.1	713	5
25	37	8.5	Hypothetical protein, Conserved	F	XP_003859244.1		5
26	37	8.5	DNA polymerase epsilon catalytic subunit	F	XP_003865021.1	258	5
27	37	8.5	GTP binding protein, putative	F	XP_003865105.1	42	1
28	37	8.5	Tyrosyl or methionyl t-RNA synthetase like protein	F	XP_003859804.1	19	1
29	24.5	8.5	Hypothetical protein	G	XP_003860609.1	347	3
30	24.5	8.5	Hypothetical protein, Conserved	G	XP_003865085.1	732	1
31	25.7	7.7	Hypothetical protein, Conserved	H	XP_003858662.1	95	4
32	23	6.2	Hypothetical protein, Conserved	I	XP_003860028.1	82	3
33	23	6.2	Dynein heavy chain, cytosolic, putative	I	XP_003860841.1	621	5
34	23	6.2	Dynein heavy chain, cytosolic, putative	I	XP_003860841.1	622	6
35	23	6.2	Dynein heavy chain, cytosolic, putative	I	XP_003859985.1	91.8	3
36	23	6.2	Putative serine/threonine kinase	I	XP_003858539.1	33	2
37	23	6.8	Dynein heavy chain, axonemal, putative	J	XP_003864528.1	528	6
38	23	6.8	Dynein heavy chain, axonemal, putative	J	XP_003864528.1	528	5

### 3.6. Validation of the presence of *L*. *donovani* antigen in CICs and MS derived peptides

**Fig 6:**
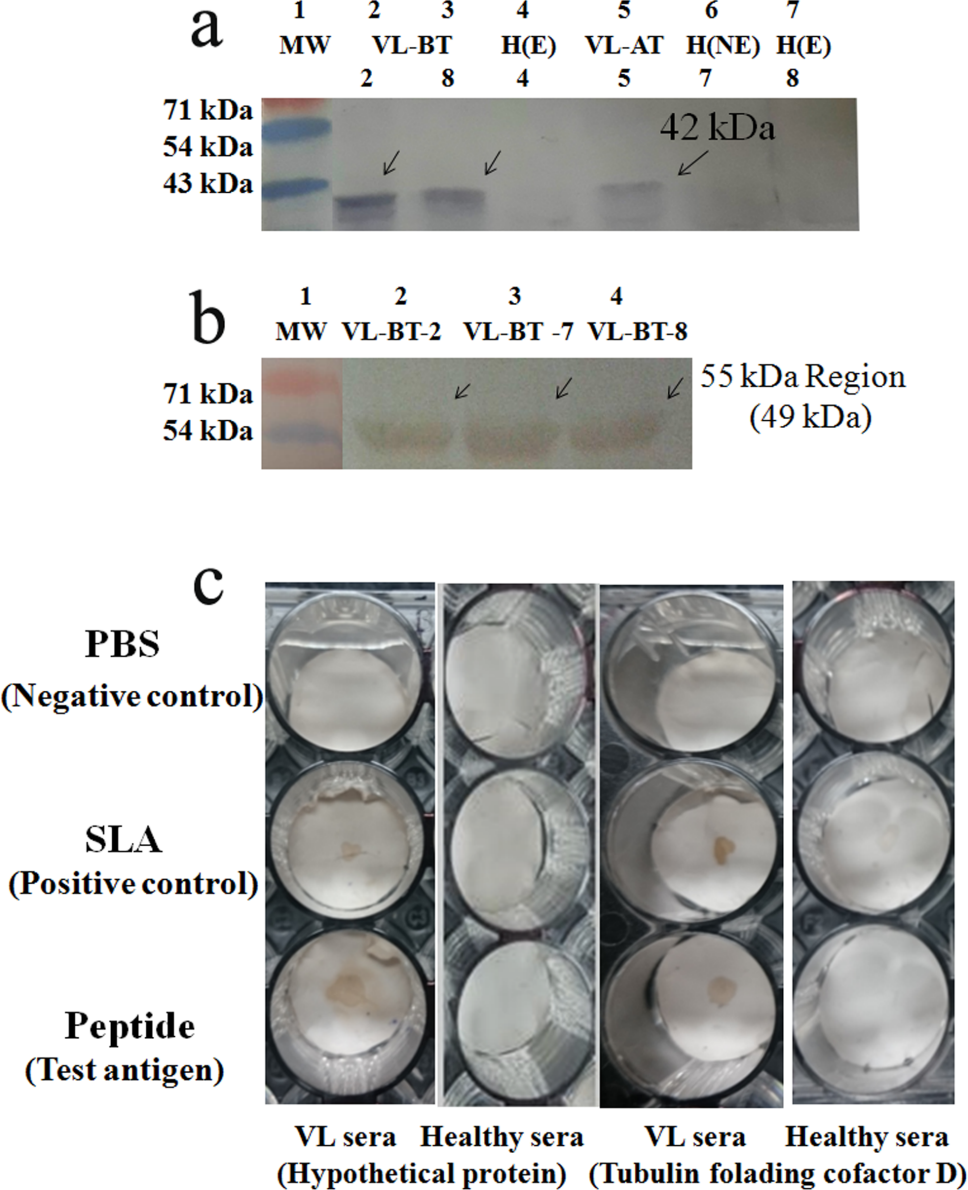
Validatory evaluation of circulating antigen. Figure showing presence of *L*. *donovani* antigen in CICs of VL-BT samples using (a) anti-*Ld* actin antibody and (b) anti-*Ld* tubulin antibody. (c) Figure showing immuno-reactivity of MS derived synthetic peptide with VL patient sera.

The Presence of anti-*Ld* actin antibody reactive against 42 kDa band was identified in VL-BT (Fig 6A). Similar band with five-fold reduction was also revealed in VL-AT subjects. However, it was absent in healthy sample. Interestingly, presence of alpha-tubulin (49 kDa) was found in 55 kDa region of CICs antigen (Fig 6B), which was in agreement with earlier report [50]. MS data was validated with spot ELISA (Fig 6C). It confirmed the reactivity of synthetic peptides with VL sera. SLA validates its *L*. *donovani* origin. Reactivity of healthy sera was negative in both cases.

## 4. Discussion

Formation of immune complexes occurs during the various scavenging process as a phenomenon of humoral immunity to remove antigen or pathogen [51]. Higher expression of the immune complex is associated with *L*. *donovani* induced inversion of the normal albumin to globulin ratio [52] and hypergamma-globulinemia in human VL subject [6, 17, 53, 54]. The Gamma-globulins trap released *Leishmania* amastigotes or its antigen and forms immune complex during various complexes forming action of humoral immune response. Usually, immune complexes are removed by scavenging macrophages. But, excessive production and defective clearance of these immune complexes lead to accumulation in circulation [55]. Previously, Evan and Pearson [17] had observed 140 fold higher accumulations of CICs in American VL subject in comparison to healthy subjects. However, it was observed 36 higher fold in the present study. This may be due to a difference in Leishmania strain of American and Indian VL as well as due to a difference in dynamics of immunity in patients. Expectedly, the higher concentration of CICs in VL subject due to hypergamma-globulinemia as reported earlier. Hypergamma-globulinemia was also reported in some systemic diseases like systemic lupus erythematosus (SLE), rheumatoid arthritis, lymphogranuloma venereum, a chronic liver disease like cirrhosis, angioimmunoblastic lymphadenopathy and some classes of lymphomas [56]. However, infectious diseases like tuberculosis, malaria, filarial, leprosy, influenza are known for comparatively lower expression of CICs [24, 31, 57, 58]. Asthma was also observed with higher expression of CICs. Anti-leishmanial therapy particularly Amphotericin B treatments improves the ratio of albuminuria to hypergammaglobulinemia, which is being used as a nonspecific biochemical parameter of prognosis in VL [59]. Anti-leishmanial therapy controls the parasite load in the patient, as well as the release of antigen in circulation. In the present study, lower expression of CICs in treated subjects is associated with lowering of parasite load as well as lowering of hypergamma-globulinemia. Though, there was statistically significant difference in concentration of CICs in study groups; this cannot be recommended for diagnosis as it is not specific only to leishmaniasis.

The thrust area to explore in this study was the identification of *Leishmania* antigen present in CICs. But the presence of higher concentration of immunoglobulins in CICs may induce shielding effect during epitope detection. Therefore, the effort was needed to minimize the concentration of immunoglobulins in samples of CICs. Previously, Gupta and Tan [46] and Hoffken *et al* [60] had successfully segregated antigens and antibodies from CICs. Methodologies applied in those studies were adopted here to minimize the concentration of immunoglobulin fragments in CICs samples. Through this, we could minimize the concentration of antibody fragments from the CICs samples.

The start-up phase of the study revealed 55 kDa and 23 kDa antigen, which showed an overexpression with consistency and immunodominance in VL-BT subjects and also showed a discriminating ability to differentiate active from a treated VL cases. Comparatively, 23kDa antigen appeared more promising diagnostic marker in comparison to 55 kDa band, which was recognizable in samples of few treated VL cases. In immunoblotting studies, the 55 kDa and 23 kDa antigen fractions were not only immunoreactive against anti-*Leishmania* polyclonal sera in samples from VL-BT; it was also present and reactive in SLA. Previously, Sanyal *et al*. [50] had also reported the presence of 55 kDa fraction in their study but not 23 kDa. Additionally, they had also confirmed the presence of the fragments of gp63, tubulin-like protein and IgG heavy chain in 55 kDa region through western blotting [50, 61].

55kDa and 23kDa fractions was absolute in VL-BT subjects. Moreover, immuno-reactivity revealed low relative intensity band in VL-AT, which was significant in some samples from treated VL subjects. Therefore, it was impossible to ascertain the prognostic value of these bands unless considering band intensity. Since one of the aims of this study was to identify an antigen of prognostic value, further segregation of antigen was needed through 2D for unambiguous characterization of circulating antigens. Additionally, above referenced studies are evident that the 55 kDa bands contain multiple peptides; it was essential to go for 2D segregation of CICs antigen samples. Since some other immunodominant 2D spots were identified in the whole antigen in addition to 2D spots segregated from 55 kDa and 23kDa; all the immunodominant spots present in whole antigens were included for further analysis. Some of the 2D spots recognized in the immunoblotting were those previously reported [50, 61, 62]. The analysis revealed of twelve immunodominant 2D spots, which were absent in healthy samples and treated samples, hence be of diagnostic value.

Though our main goal was to decode *Leishmania* antigen in 2D spots, we encrypted antigenic peptide through MS. LC-ESI-MS/MS data of the spots evaluated in the present study after analysis through search database (NCBI, UniProt, Ludwig NR database) revealed more than one protein in a single spot, for example, cytosolic dynein heavy chain, kinesin, 60 S ribosomal proteins in spot number B. There were also presences of many hypothetical conserved proteins in different spots. These proteins may be used in the diagnosis and other purposes. Peptide fractions reported from different spots were identified as hypothetical/uncharacterized Leishmania proteins along with putative kinesin of *L*. *donovani*, major surface protease gp63, putative tubulin, DNA polymerase epsilon catalytic subunit, GTP-binding protein, and tyrosyl-methionyl t-RNA synthetase-like protein. Obtained data on gp63 confirmed the reports of Chakraborty *et al*. [62] in which, they had reported fractioned gp63 at dislocated site using monoclonal antibody. Besides, tubulin was also reported earlier with the help of monoclonal antibodies [50, 61, 62]. In this study, we also recognized it through LC-ESI-MS/MS data analysis. Protein like t-RNA synthetase, ribosomal protein, DNA-directed RNA polymerase subunit, and protein kinase was also reported by Kumar *et al*. [63]. Some of the peptides reported in this study are associated with GTP-binding protein and increase in expression of 60S ribosomal subunit as well as nucleosome assembly protein. These are important parasite antigen being associated with the possible loss of infectivity of *L*. *amzonensis* [64]. Madalhaes et al, 2014 had reported the decrease in expression level of these proteins in SLA [65]. Whereas, the expression of all the three proteins was increased. The presence of highly conserved ribosomal protein, proteins of the DNA replication and transcription identified in the samples of VL-BT is confirmed by the earlier finding of Requena *et al*. [66].

In fact, uncharacterized hypothetical peptides of unknown function were observed in high concentration. Kumar *et al*. [63] had reported the presence of 22% hypothetical proteins in SLA from *L*. *donovani*. Likewise in the present study, 50% hypothetical protein is being reported in CICs of VL-BT subjects. These proteins could be of prime concern understanding functional aspect, their role in pathogenicity, drug resistance, disease control and intracellular survival. Future, these novel proteins can be proved crucial not only for diagnosis but also for potential drug target, vaccine development, and therapeutics. Most of the reported proteins of known function are associated with physio-pathology. Other identified peptides were from motor proteins, proteins involved in phosphorylation and synthesis, cytoskeleton organization, carbohydrate metabolism, transcription, DNA replication, cell adhesion, signal transduction and nuclear assembly.

This study is reporting the presence of *L*. *donovani* specific antigens in CICs of VL-BT subjects. Previously, several diagnostic kits viz. rapid Dengue NS-1 antigen test [30, 67, 68], P24 HIV test [69], Carsinoembryonic antigen test (CEA test) [70], HRP2 antigen test [71, 72] etc. have been successfully developed targeting immune complex antigens. Due to highly perishable nature of protein antigen and due to scavenging and salvage process, antigens disappear or decrease in the subject receiving anti-leishmanial therapy. The residual amount of antigen also disappears quickly in comparison to antibodies from treated subjects. The presence of the *Leishmania* antigen may be detected in peripheral blood using antibodies which will be reverse to the existing RDT.

In this respect, we further validated the MS derived peptide for its reactivity with patience in section 3.6. These synthetic peptides revealed reactivity with VL sera, but not with healthy sera. However, the presence of *L*. *donovani* antigens in CICs (Fig 6A & 6B) further strengthen the *L*. *donovani* origin of circulating antigen. Earlier, presence of anti-actin and anti-tubulin antibody was also reported [50, 73,74]. Hypothetical protein (XP_003861271.1, spot C) successfully shown the efficiency of targeted MS derived peptide through Immuno chromatographic test (ICT) [9]. Above protein is under process of translational research for application. In another attempt, peptide from known protein of *L*. *donovani* i.e. tubulin folding cofactor D from spot E (AccNo.XP_003861300.1, spot D, under review) was characterized employing antigen capture ELISA. Therefore, the peptides reported in the present study can be used for development of diagnostic as well prognostic kit.

## Supporting information

S1 Fig Figure showing the eluted 23 kDa and 55 kDa antigen band. CBB stained 10% SDS-PAGE showing lane-1: molecular weight standard, lane-2: 55kDa antigen and lane-3: 23kDa antigen.
(TIF)

S2 Fig **Figure showing silver stained 55 kDa(A) and 23 kDa (B) antigens dissociated into several 2D spots.** 2D induced further fractionation in 55 kDa (Fig 2A) and 23 kDa fraction (Fig 2B) isolated from SDS-PAGE gel.
(TIF)

S3 Fig Figure showing the absence of silver-stained 2D spots at pH 8.5 in VL-AT subjects in comparison to VL-AT. 40μg of proteins was subjected to IPG strips (3–10 pH, 4–7 cm, non-linear) in the first dimension followed by SDS-PAGE (12%) in the second dimension.
(TIF)

S4 Fig Figure showing densitometry (volume, concentration and area) study of silver stained 2D gel. (a) Comparison on the basis of spot intensity between two dimensional electrophoresis of antigens of (A) VL-BT subject and (B) healthy subject. (b) Figure showing graphical representation of densitometry analysis of silver stained 2D spots of VL-BT subjects and healthy subject.
(TIF)

S1 Table Table showing molecular weight (kDa) and isoelectric point (pI) of silver-stained 2D spots in VL-BT and healthy subjects. Calculation was done using Quantity One software.
(DOCX)

S2 Table Table showing relative intensity (mm^2^) of silver-stained 2D protein spots in VL-BT and healthy subjects. Calculation was done using Quantity One software.
(DOCX)

S3 Table Table showing relative intensity (mm^2^) of immunoreactive 2D protein spots in VL-BT and healthy subjects. Calculation was done using Quantity One software.
(DOCX)

S1 Dataset Data showing screen shots of mass spectrometry data. Snap shot revealed protein hits of all the 2D spots.
(DOCX)
