# Supplementary figures and images for: Identification of Leishmania donovani antigen in circulating immune complexes of visceral leishmaniasis subjects for diagnosis
Source: PLoS One. 2017 Aug 18;12(8):e0182474. doi: 10.1371/journal.pone.0182474 (PMC5562322; doi:10.1371/journal.pone.0182474)

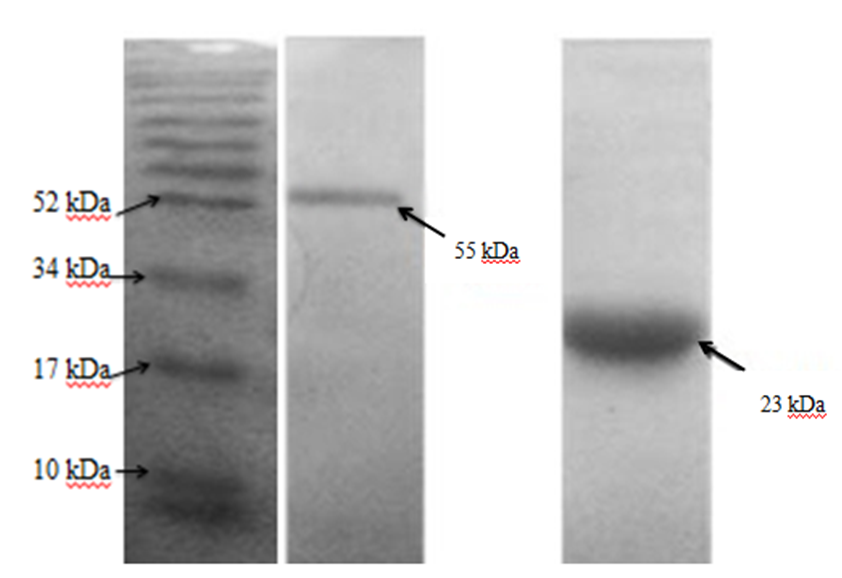

Supplement: S1 Fig — CBB stained 10% SDS-PAGE showing lane-1: molecular weight standard, lane-2: 55kDa antigen and lane-3: 23kDa antigen. (TIF) [file pone.0182474.s001.tif]

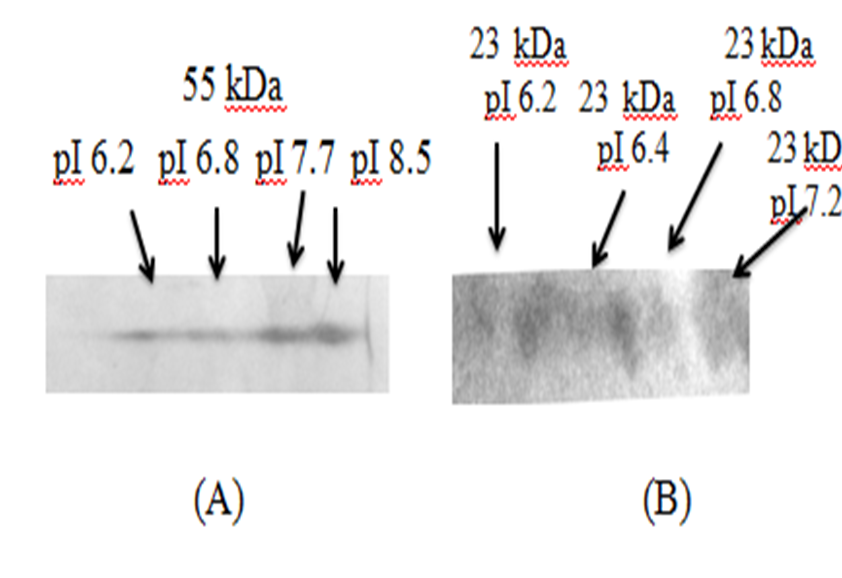

Supplement: S2 Fig — Figure showing silver stained 55 kDa(A) and 23 kDa (B) antigens dissociated into several 2D spots. 2D induced further fractionation in 55 kDa (Fig 2A) and 23 kDa fraction (Fig 2B) isolated from SDS-PAGE gel. (TIF) [file pone.0182474.s002.tif]

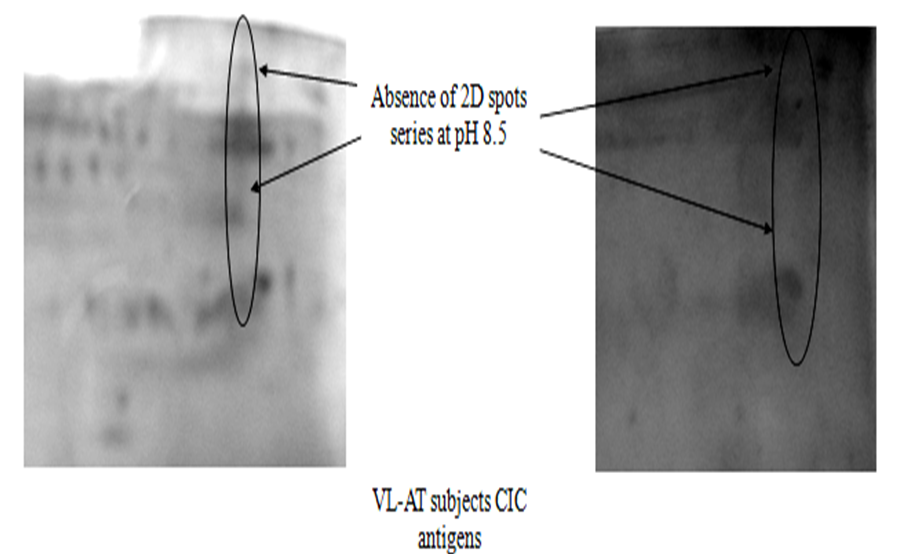

Supplement: S3 Fig — 40μg of proteins was subjected to IPG strips (3–10 pH, 4–7 cm, non-linear) in the first dimension followed by SDS-PAGE (12%) in the second dimension. (TIF) [file pone.0182474.s003.tif]

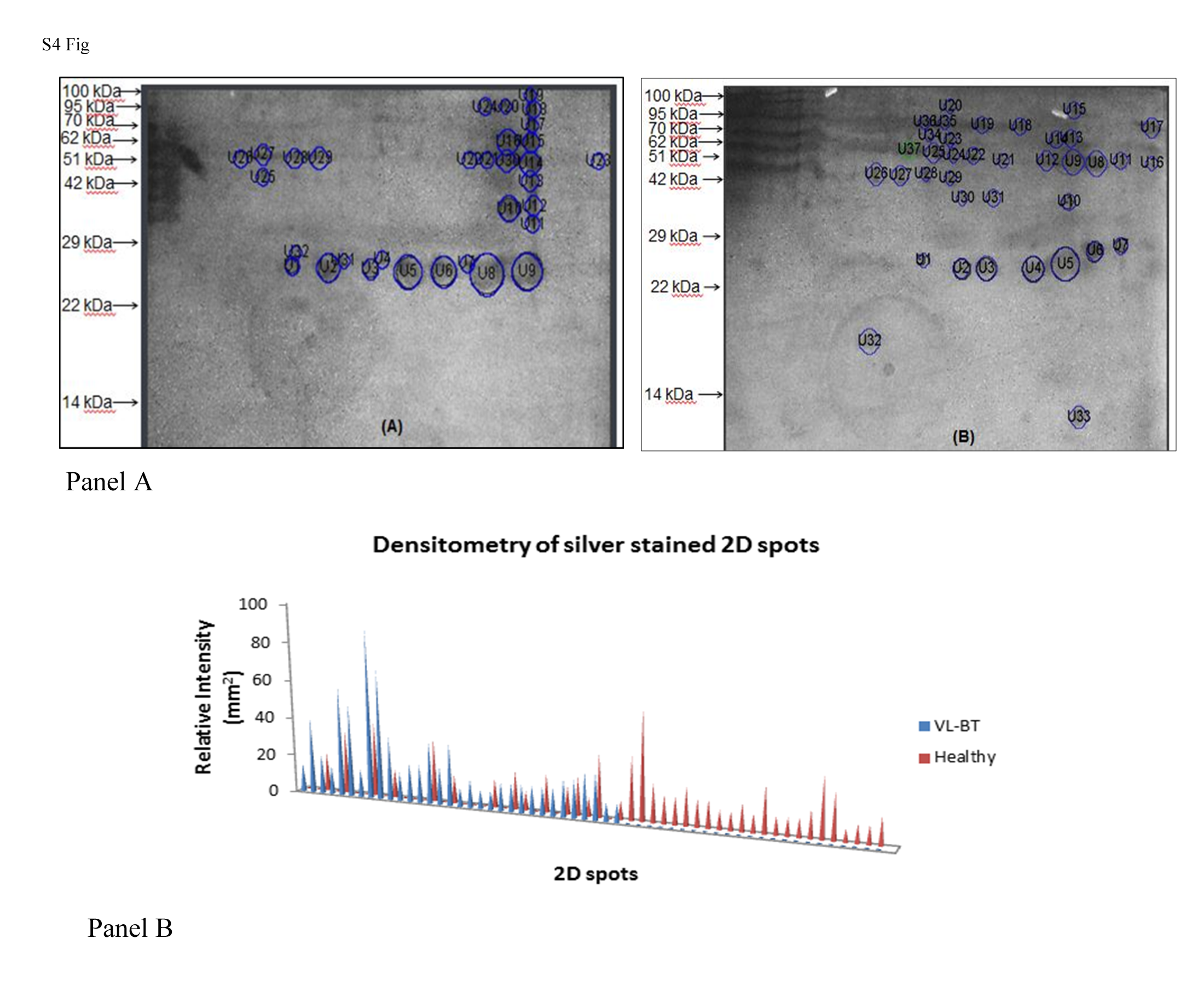

Supplement: S4 Fig — (a) Comparison on the basis of spot intensity between two dimensional electrophoresis of antigens of (A) VL-BT subject and (B) healthy subject. (b) Figure showing graphical representation of densitometry analysis of silver stained 2D spots of VL-BT subjects and healthy subject. (TIF) [file pone.0182474.s004.tif]

**S1 dataset**


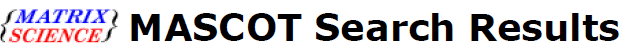


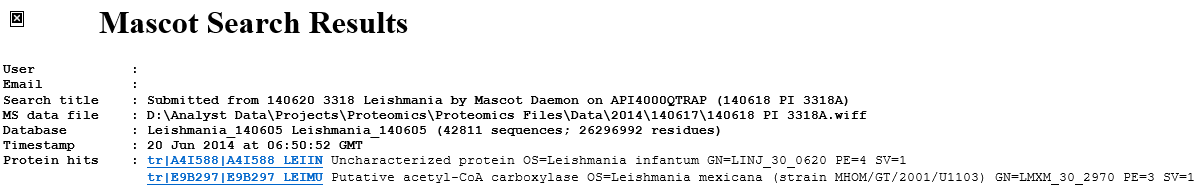


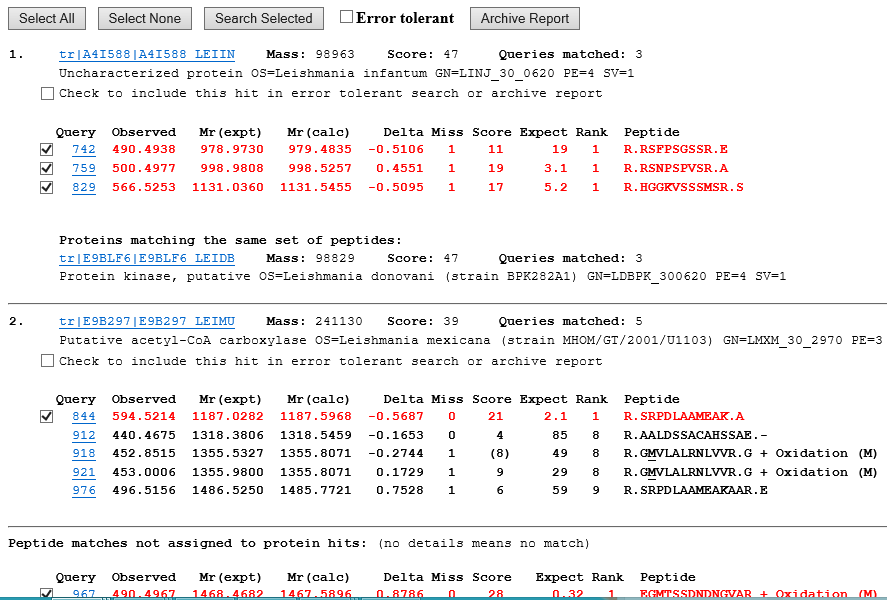


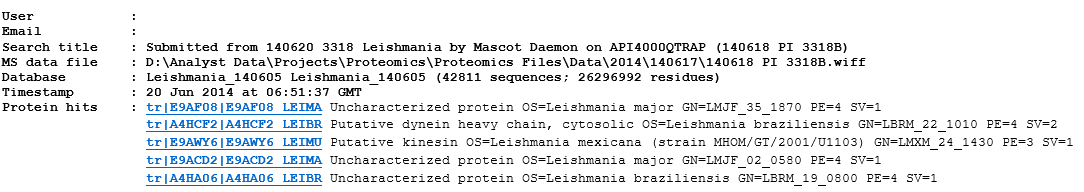


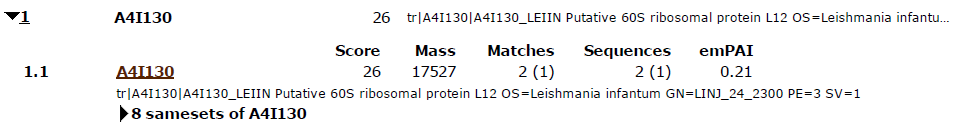


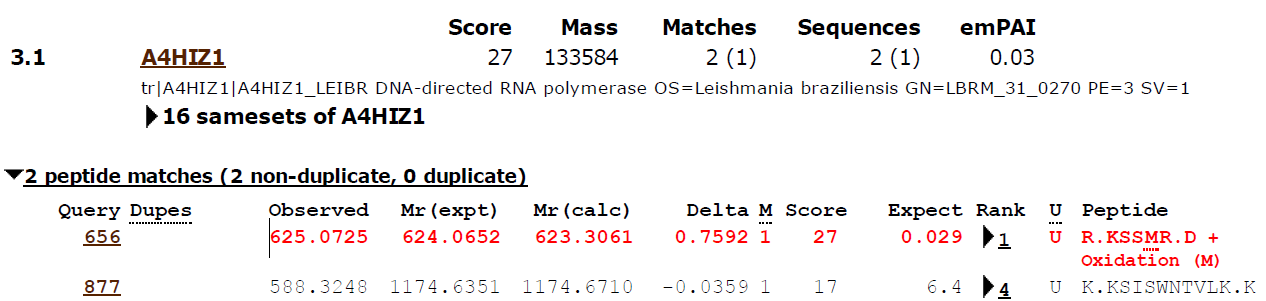


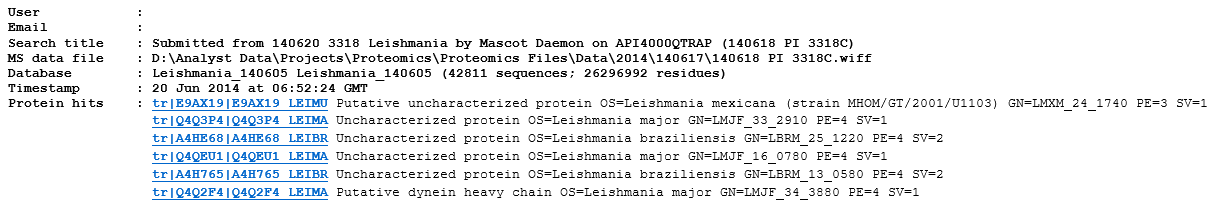


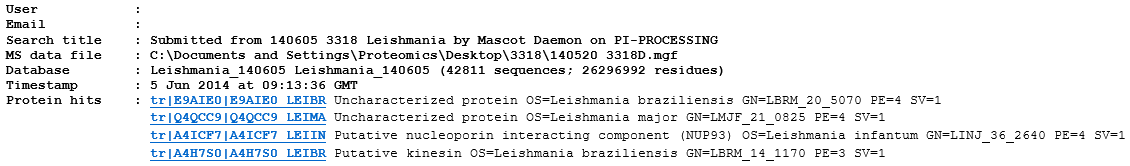


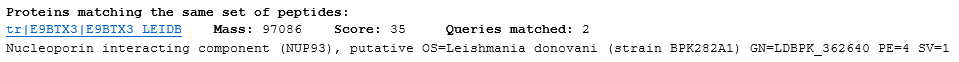


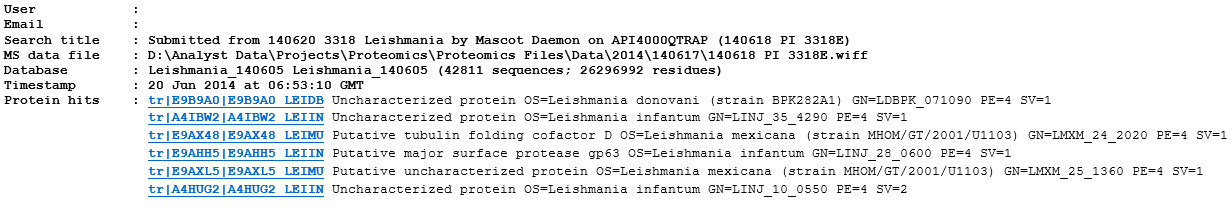


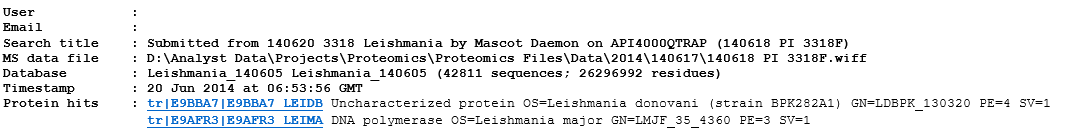


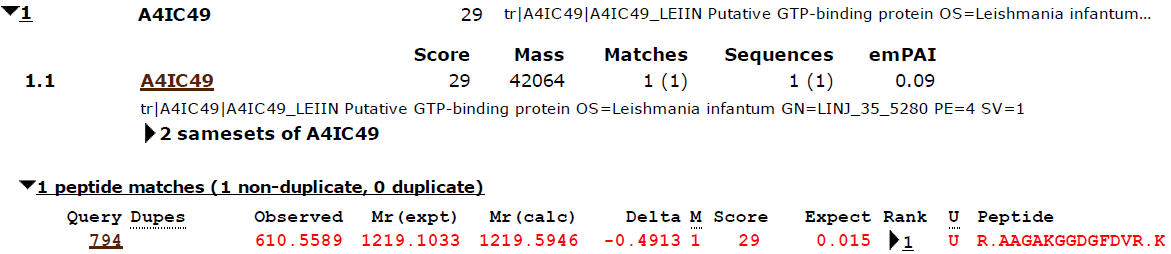


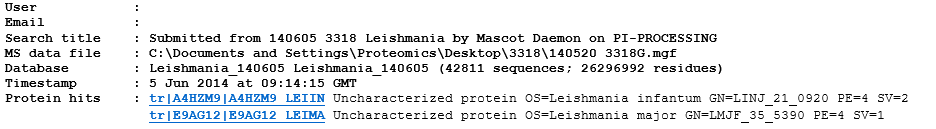


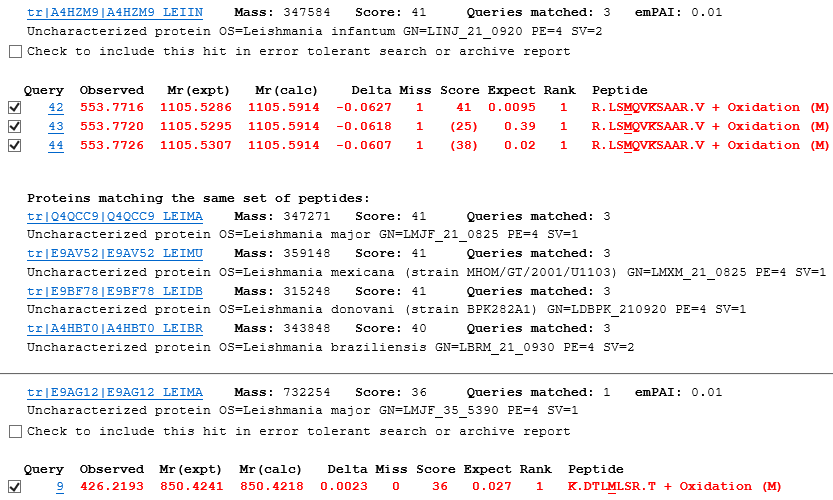


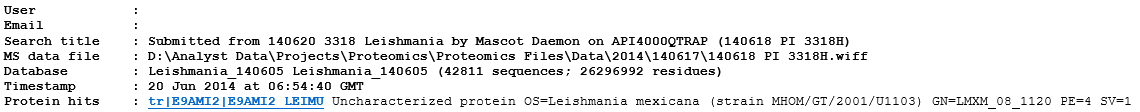


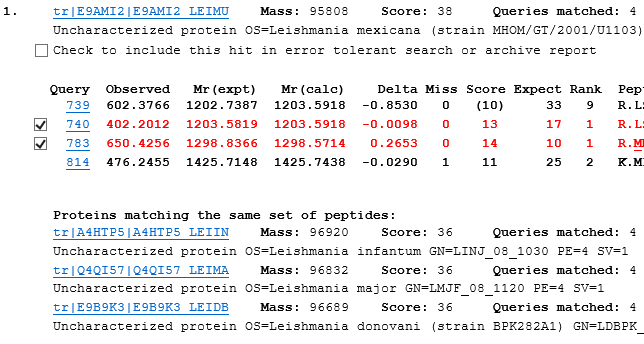


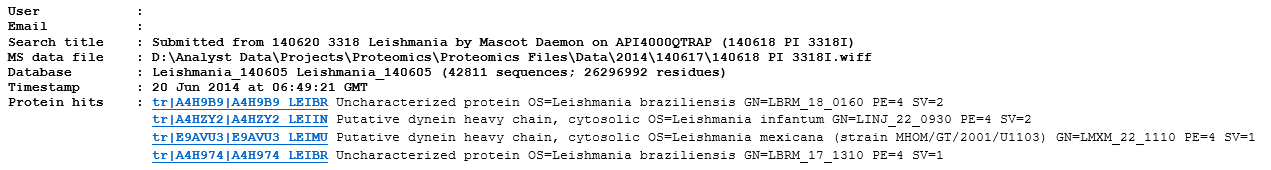


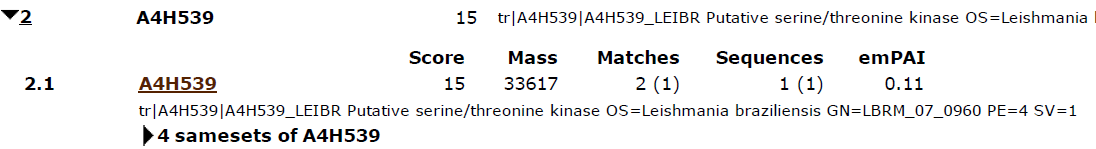


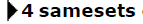

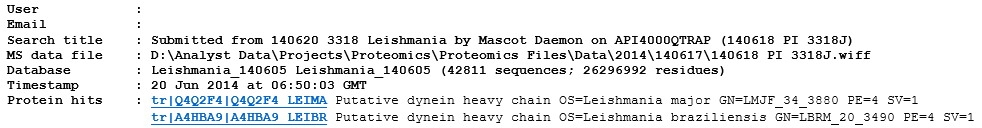

Supplement: S1 Dataset — Snap shot revealed protein hits of all the 2D spots. (DOCX) [file pone.0182474.s008.docx]
