# Supplementary material for: Identification of Leishmania donovani antigen in circulating immune complexes of visceral leishmaniasis subjects for diagnosis
Source: PLoS One. 2017 Aug 18;12(8):e0182474. doi: 10.1371/journal.pone.0182474 (PMC5562322; doi:10.1371/journal.pone.0182474)
Supplement: S1 Table — Calculation was done using Quantity One software. (DOCX) [file pone.0182474.s005.docx]

**S1 Table**

S1 Table. Table showing molecular weight (kDa) and isoelectric point (pI) of silver-stained 2D spots in VL-BT and healthy subjects. Calculation was done using Quantity One software.

| Different study group | VL-BT subjects | Healthy subjects |
| --- | --- | --- |
| Molecular weight and Isoelectric point of silver stained antigenic 2D spots | 125 kDa ( pI 8.5), 100 kDa ( pI 8.5), 93.3 kDa ( pI 8.5), 87 kDa ( pI 8.5, 7.7,6.8), 79.4 kDa ( pI 6.6, 6.4, 6.2, 6), 67.6 kDa ( pI 8.5), 67.5 kDa (pI 8.5), 63 kDa (pI 8.5, 7.7), 60.2 kDa (pI 5.8, 5.6, 5.3, 5 ), 55 kDa ( pI 8.5, 7.7, 7.6, 7.5, 6.8, 6.2, 6) , 45 kDa ( pI 8.5), 43.6 kDa ( pI 5.8, 5.6, 5.3, 5), 41.6 kDa ( pI 8.5), 39.8 kDa (pI 7.7), 37 kDa ( pI 8.5), 26.3 kDa ( pI 6), 25.7 kDa ( pI 7.7, 6.3, 5.8, 5.5), 24.5 kDa (pI 8.5, 6.5, 5.9, 5.7, 5.3), 23 kDa (pI7.2, 6.8, 6.4, 6.2) and 20 kDa (pI 8.5) | 87 kDa (pI 7.7), 79.4 kDa (7.7, 6.8, 6.6, 6.4, 6.2, 6), 63 kDa (pI 9.7, 7.7), 60.2 kDa (pI 6, 5.8, 5.6, 5.3, 5), 55 kDa (pI 9.7, 9.2, 7.7, 7.6, 7.5, 6), 43.6 kDa (pI 5.8, 5.6, 5.3, 5), 41.6 kDa ( pI 7.7, 6.5, 6.2), 39.8 kDa (7.7), 28 kDa (pI 9.7, 9.2, 8.5), 26.9 kDa (pI 5.7, 8, 8.3), 26.3 kDa (pI 8, 7.3, 6.5, 6.2, 6), 25.7 kDa (pI 9.7, 9.2, 7.6, 5.8, 5.3), 23 kDapI7, 7.7, 19 kDa (pI 5.2), 15 kDa (pI 7.7) and 11 kDa (pI 8) |
