# Supplementary material for: Identification of Leishmania donovani antigen in circulating immune complexes of visceral leishmaniasis subjects for diagnosis
Source: PLoS One. 2017 Aug 18;12(8):e0182474. doi: 10.1371/journal.pone.0182474 (PMC5562322; doi:10.1371/journal.pone.0182474)
Supplement: S2 Table — Calculation was done using Quantity One software. (DOCX) [file pone.0182474.s006.docx]

**S2 Table**

| Different study group | VL-BT subjects | Healthy subjects |
| --- | --- | --- |
| Relative density of silver stained 2D protein spots | (U1-U32) in INT*mm^2^ in case of VL-BT subjects were 2490.9, 6492.7, 3152.6, 1973.3, 8854.6, 7191.3, 2055, 12671.4, 10076.8, 5804.6, 2209.7, 3192.3, 3249.6, 5767.2 , 3724.4, 5976.5,1822.8, 2554, 1767, 1654, 2462.4, 2119, 1758.2, 2343.1, 2190.6, 2200.1, 3183.8, 2994.5, 3552.2, 4433.9, 1499 and 1492. Relative intensity in term of area of these (U1-U32) spots in mm^2^ were 15, 39.9, 20.5, 15, 58.8, 48.9, 15, 90.2, 68.8, 33.4, 15, 20.5, 20.5, 33.4, 20.5, 33.4, 10, 15, 10, 10, 15, 15, 15, 15, 15, 15, 20, 20, 25, 25, 10 and 10. | (U1-U37) in INT*mm2 in case of healthy subjects were 1351.1, 2697.2, 4701.9, 4551, 8136.8, 3484.7, 2387.1, 5128.4, 5611.2, 2145, 3296.8, 2619.2, 2578.2, 2469, 1295, 3065.5, 2512.4, 2592.2, 1640, 1463.8, 2388.3, 1662.3, 1605.5, 3269.7, 3356.8, 2776.6, 1545, 1480.1, 1332, 1937.2, 3559.3, 2822, 1130.7, 1761.9, 1795.8 and 2480.3. Relative intensity in term of area of these spots (U1-U37) in mm^2^ were 10, 20.5, 33.4, 33.4, 58.8, 20.5, 15, 33.4, 33.4, 15, 15, 20.53, 15, 15, 15,10, 20.5, 15, 15, 10, 10, 15, 10, 10, 20.5, 25.5, 20.5, 10, 10, 10, 15, 33.4, 25.5, 7, 10, 10 and 15. |

S2 Table. Table showing relative intensity (mm^2^) of silver-stained 2D protein spots in VL-BT and healthy subjects. Calculation was done using Quantity One software.
