# Supplementary material for: Identification of Leishmania donovani antigen in circulating immune complexes of visceral leishmaniasis subjects for diagnosis
Source: PLoS One. 2017 Aug 18;12(8):e0182474. doi: 10.1371/journal.pone.0182474 (PMC5562322; doi:10.1371/journal.pone.0182474)
Supplement: S3 Table — Calculation was done using Quantity One software. (DOCX) [file pone.0182474.s007.docx]

**S3 Table**

S2 Table. Table showing relative intensity (mm^2^) of immunoreactive 2D protein spots in VL-BT and healthy subjects. Calculation was done using Quantity One software.

| Different study group | VL-BT subjects | Healthy subjects |
| --- | --- | --- |
| Relative density of immunoreactive 2D protein spots | (U1 to U12)  4821INT*mm^2^ (29.8 mm^2^), 5205.4 INT*mm^2^ (33 mm^2^), 4709 INT*mm^2^ (29 mm^2^), 5679.2 INT*mm^2^ (36.8 mm^2^), 2448.2 INT*mm^2^ (13.5 mm^2^), 2931.8 INT*mm^2^ (15.8 mm^2^), 5903 INT*mm^2^ (33.2 mm^2^), 3942.6 INT*mm^2^ (20.7 mm^2^), 4107.7 INT*mm^2^ (20.7 mm^2^), 4859.1 INT*mm^2^ (23.7 mm^2^), 5594.6 INT*mm^2^ (26.6 mm^2^), 108 INT*mm^2^ (0.8 mm^2^) | (U1 to U7)  3477.4 INT*mm^2^ (26.6 mm^2^), 1627.5 INT*mm^2^ (15.8 mm^2^), 2105.4 INT*mm^2^ (20.7 mm^2^), 3001.5 INT*mm^2^ (29.8 mm^2^) and 1105 INT*mm^2^ (11.2 mm^2^), 1200.9 INT*mm^2^ (15.8 mm^2^), 1713 INT*mm^2^ (23.77 mm^2^) |
